# Evaluation of the Effectiveness of a Musical Cognitive Restructuring App for Black Inner-City Girls: Survey, Usage, and Focus Group Evaluation

**DOI:** 10.2196/11310

**Published:** 2019-06-27

**Authors:** Angela Neal-Barnett, Robert Stadulis, Delilah Ellzey, Elizabeth Jean, Tiffany Rowell, Keaton Somerville, Kallie Petitti, Benjamin Siglow, Arden Ruttan, Mary Hogue

**Affiliations:** 1 Kent State University Department of Psychological Sciences Program for Research on Anxiety Disorders among African Americans Kent, OH United States; 2 Kent State University Department of Computer Science Kent, OH United States; 3 Kent State University College of Business Kent, OH United States

**Keywords:** black girls, musical cognitive restructuring, mHealth, negative thinking

## Abstract

**Background:**

Research on mobile health (mHealth) app use during adolescence is growing; however, little attention has been paid to black adolescents, particularly black girls, who are generally underresearched and underserved in psychological intervention research. Cognitive restructuring is an important tool in anxiety and fear management and involves two parts: (1) recognizing and deconstructing erroneous thoughts and (2) replacing negative anxiety and stress-provoking thoughts with positive thoughts. In our work with black adolescent females, we found that cognitive restructuring is a difficult skill to practice on one’s own. Thus, drawing upon the importance of music in the black community, we developed the Build Your Own Theme Song (BYOTS) app to deliver a musical form of the technique to middle-school black girls.

**Objective:**

Our aim in this mixed methods study is to evaluate the effectiveness of the BYOTS app. We hypothesize that participants will expect the app to be effective in reducing negative thoughts and that the app will meet their expectations and data generated from the app will demonstrate a reduction in negative thinking and anxiety.

**Methods:**

A total of 72 black or biracial seventh- and eighth-grade adolescent females were enrolled in Sisters United Now (SUN), an eight-session culturally infused and app-augmented stress and anxiety sister circle intervention. Before using the BYOTS app, girls completed the Multidimensional Anxiety Scale for Children 2 and the App Expectations Survey. Usage data collected from the app included an assessment of negative thinking before and after listening to their song. After completion of the intervention, focus groups were held to gather qualitative data on participants’ app experience.

**Results:**

Results using paired sample *t* tests indicated negative thinking was significantly lower at day 7 than day 1 (*t*_31_=1.69, *P*=.05). Anxiety from preuse to postuse of the app was also reduced (*t*_38_=2.82, *P*=.004). Four effectiveness themes emerged from the focus groups: difference in behavior and temperament, promoted calmness, helpfulness in stressful home situations, and focused thinking via the SUN theme song.

**Conclusions:**

The BYOTS app is a useful tool for delivering musical cognitive restructuring to reduce negative thinking and anxiety in an underserved urban population. Changes were supported both quantitatively and qualitatively. Participants, their peers, and their family noted the difference. Findings support expanding the research to black girls of various socioeconomic statuses and geographic diversity. Currently, the app augments SUN, a culturally relevant intervention. Future research will explore BYOTS as a stand-alone app.

## Introduction

### Background

Anxiety is a common problem among adolescents and has an adverse impact on their lives. Anxiety symptoms are associated with poor academic performance [1,2], difficulty with social skills and peer relationships [3,4], and behavior difficulties [5]. Several studies have suggested racial differences in symptoms and intensity of anxiety in youth. Compton and colleagues [6] found that black youth reported significantly more harm avoidance and physical avoidance than their white peers. Latzman and colleagues’ [7] study of depression and anxiety across three cohorts of youth—elementary, middle school, and high school—found higher anxiety symptoms in middle school.

### Anxiety and Black Females

Anxiety symptoms for black females are chronic and more intense [8]. Regularly elevated anxiety levels are reported for black girls residing in urban areas across the United States [9-12]. Research indicates that, if left unaddressed, anxiety difficulties in adolescence are associated with a higher risk for anxiety disorders as adults [13,14]. Black women with higher levels of anxiety also report poor emotional regulation [13]. Given the elevated anxiety levels among urban black girls [9-12], the intensity and chronicity of anxiety symptoms [8], and the implications for adulthood [13,14], early intervention with this population is appropriate. Yet the literature is clear as it relates to anxiety and other mental health issues: black girls are underresearched and underserved [15,16]. Reasons for this include, but are not limited to, cultural mistrust, access to mental health care, access to same-race mental health care, and stigma [15,16].

### mHealth and Adolescents

A plethora of mHealth apps exist for anxiety, but few are evidence-based, and even fewer are specifically designed for adolescents [17,18]. Nonetheless, the available literature suggests that given adolescents’ frequent use of mobile phones, mHealth apps can serve as effective forms of early intervention or augmentations to more traditional forms of intervention [19]. This technology may be particularly helpful for underserved black adolescents as an early anxiety intervention because 81% of this population reported using a mobile phone on a daily basis [20,21].

### Cognitive Restructuring

Cognitive restructuring is an important tool in anxiety management [22]. Operationally defined, cognitive restructuring involves two parts: (1) recognizing and deconstructing erroneous thoughts and (2) replacing the negative anxiety and stress-provoking thoughts with positive thoughts [22]. In our work with black females, we have found at times that cognitive restructuring is a difficult skill to practice on one’s own [23]. Outside of the therapy setting, our clients reported that they found it “too hard” and that they “couldn’t do it,” suggesting there may be a need for a culturally informed technique to assist in the steps necessary to teach and execute cognitive restructuring. One way to do so is to merge the technique with music.

### Black Adolescents and Music

Music is an important part of almost every adolescent’s life [24,25]. The tempo, genre, rhythm, and lyrics combine to allow youth to express thoughts and feelings they may not be able to verbalize in other ways [25]. Female adolescents are more likely to use music to express their emotional state than their male counterparts [26]. Unlike adolescents of previous eras, mobile phone technology allows today’s youth to take their music everywhere they go [20].

For black youth, music is a powerful tool for expressing and overcoming emotion [23,27-32]. Over the last 70 years, songs have been an integral part of the black youth experience, providing hope, empowerment, faith, and encouragement [23,27-32]. Reflecting on the importance of spirituals and gospel music to black adolescents and emerging adults during the civil rights era, icon Euvester Simpson said, “The only thing that got us through that...we sang. We sang all night. I mean songs got us through so many things, and without that music, I think many of us would have just lost our minds or lost our way completely” [30]. The importance of gospel music in stressful situations still resonates today. A recent study with black young adults found many used gospel songs with instructive lyrics when stressed [31].

The expression of emotion and feelings is not limited to religious music. Hip-hop emerged from low-income urban areas to give a voice to black youth who were disenfranchised and marginalized [23]. As a result, for the first time, their experiences and concerns were validated and heard [23,32]. Although the commercial success of the genre co-opted the topics of the rhymes, a recent resurgence has resulted in a return to lyrics reflecting social justice, racism, and inequality issues of concern to black youth [23]. Indeed, recent research suggests that a component of hip-hop, rap, is the perfect form of music therapy for black adolescents [32].

Building on the evidence that music provides a voice to the emotions and experience of females [1,2,33] and black adolescents [23,27-32], and the potential impact of mHealth apps on the emotional lives of adolescents [19,21], we developed the Build Your Own Theme Song (BYOTS) app [33,34]. Whereas other researchers have used cognitive restructuring and music separately within the same intervention with black African couples [35], in our work we have integrated music directly into the cognitive restructuring process. Rather than developing positive statements to replace negative thoughts, our participants are taught to build their own theme song with positive lyrics to counteract negative thoughts. In our intervention studies, when a black female adolescent pays attention to her thoughts and recognizes that she is thinking negatively, she uses her theme song to counteract and replace those thoughts with positive thoughts.

### Build Your Own Theme Song App

The BYOTS mHealth app consists of a self-penned, self-recorded theme song based on the girl’s favorite song. Consistent with the research that shows mHealth apps for anxiety have the greatest impact when augmenting a face-to-face intervention [17], the BYOTS mHealth app is integrated into an eight-session culturally infused Sisters United Now (SUN) sister circle that takes place during the school day [11,33,34]. SUN and the app were developed to address the neglect of black adolescent girls in anxiety-related interventions [11,15]. During the intervention, girls are introduced to the negative and positive thought cycles. They learn how a theme song can interrupt the negative thought cycle by replacing the negative thoughts with positive affirming thoughts. Within our work, we have labeled this process as musical cognitive restructuring. The girls first choose their favorite song. As the intervention takes place in schools, in accordance with school rules, the song must not contain profanity. They then use a vision statement that was generated earlier in the intervention and a positive word bank to rewrite their favorite song into their own personal theme song. After the lyrics are revised, the new theme song is recorded on the BYOTS app.

An additional feature of the app is the pre-post app survey. When participants open the app, the survey is presented. Based on the survey score, the girl is instructed to play her theme song. After the song is completed, the survey appears again. Based on the survey score, the girl either receives a positive message praising her for changing her thinking or instructing her to play her theme song again. Three push alerts per day (before school, after school, and at night) are built into the app to prompt girls to use it multiple times per day. The girls are also instructed to use the app without prompts when they experience negative thinking.

In this mixed methods study, we present data on the effectiveness of the BYOTS app. Although a repeated measures approach to measure the participants’ evaluation of the app’s effectiveness would be desirable, a preassessment of their app expectations was only possible. Specifically, we predict that participants would expect the app to be helpful in reducing negative thoughts and that, after working with the app, their positive expectations will be met. To test this prediction, we examine girls’ expectations of the app and compare these to their experience after using their app. Specifically, we examine the ease of using the app, including recording their theme song. We also looked at their comfort using the app in various settings and with others (family and friends) present. We also hypothesize that using the app will reduce negative thinking. To test this hypothesis, evidence of negative thinking was examined on day 1 and day 7 of the 1-week app experience; we predict that the presence of negative thinking will be lower at the end of day 7. Anxiety was also assessed before and after the app-augmented SUN experience; we predict that anxiety will be reduced after the SUN intervention.

## Methods

### Materials

The Multidimensional Anxiety Scale for Children 2 (MASC-2) [35] is a 50-item instrument that assesses anxiety symptoms using a four-point Likert-like scale from 0 to 3 (0=never, 1=rarely, 2=sometimes, and 3=often) in children. It contains items such as “I worry about other people laughing at me” and “I have trouble asking other kids to play with me.” Total anxiety score was used to assess overall anxiety symptom level. This measure has been found to have good reliability (Cronbach alpha=.90) and good convergent validity [11,12]. Additionally, this measure has been found to be reliable in our sample (Cronbach alpha=.87) [11,36].

#### Pre and Post In-App Survey

This survey consists of five items. The first statement assesses general stress level, and the remaining four questions assess negative thought. Questions about negative thought included (1) I have little control over important things in my life, (2) I am confident in my ability to handle personal problems, (3) things are not going my way, and (4) only good things lie before me. Two items are reverse coded. The survey uses a seven-point Likert scale (1=strongly disagree, 2=disagree, 3=slightly disagree, 4=neither, 5=slightly agree, 6=agree, 7=strongly agree). All app responses were uploaded to a secure online server. For each entry, responses were combined for negative thought total score. A score greater than 16 on the four negative thinking questions (the median score on the scale that could range from 4 to 28) resulted in a prompt to listen to their theme song. On both days 1 and 7, the first survey response of the day was used in the analysis. If there was only one survey response for the day, this entry was retained for the analysis. Ellzey’s recent results supported the validity of the In-App Survey [33].

#### The Expectations of App Survey

This measure consisted of 14 questions on a seven-point Likert scale and was used specifically to measure girls’ expectations of the BYOTS mobile app prior to the start of the intervention. Sample questions included, “It will be practical to pull out my phone and use the app when I feel stressed” and “I believe the app will be useful in calming my nerves for specific situations when I feel stressed.” Higher scores indicated more positive expectations. A Cronbach alpha coefficient was calculated (Cronbach alpha=.893) indicating the survey was reliable for this sample.

#### Theme Song Building Session Evaluation

Administered after the completion of the theme song lyrics and recording, this five-item evaluation examined girls’ satisfaction with using the app to build and record their theme song. Sample statements included “I enjoyed recording my own theme song” and “Practicing using the app during the session helped me learn.” Each item was rated on a five-point scale from strongly disagree (1) to strongly agree (5).

#### Guided Discussion Focus Group Transcripts

At the end of the intervention, guided discussions to gain insight into participants’ experiences with the app were held. These discussions were audio recorded and transcribed. Open-ended questions were posed, such as “What made it easy to use? What made it hard?” and “Has using the app changed the way you think about yourself?” All participants were encouraged to provide their perceptions on how helpful the app was in changing their negative thinking. Audio recordings of the six focus discussions were transcribed.

### Participants

Participants were 72 black or biracial seventh- and eighth-grade adolescent females. However, the sample size for each analysis varied due to initial connectivity issues between the app and the secure server in which data were unable to be matched to the participant. Participants were between the ages of 12 and 15 years and enrolled in the SUN program. Participants attended one of two middle schools located in a large midwestern, low-income, urban school district in the United States. All students within the district were enrolled in the federal free breakfast and lunch program. Informed consent was obtained from both the girls and their parent(s) or guardian(s). Participants either self-selected to participate or were recommended for SUN by their school counselor. The study was approved by the Kent State University Institutional Review Board, Kent, Ohio.

### Procedure

As part of the SUN intervention, girls recorded their theme songs into the app and practiced using the app in-session. The girls were then prompted three times throughout the day for a 1-week period to use the app. Data on app usage were collected in real time. On their return to SUN, a focus group was led by a third-party individual to assess the girls’ experiences with the app.

### Statistical Analysis

Quantitative data was analyzed using SPSS. The following App Expectations Survey items were chosen to test our predictions about the app’s effectiveness: “Girls will find the app helpful” (item 8), “I expect that after using the app I will feel less anxious in general” (item 9), “The app makes me feel powerful” (item 10), and “I want to use the theme song app” (item 14).

At the end of the intervention, girls completed an evaluation, which included an item to assess their perception of the effectiveness of listening to their song to help reduce their negative thinking. To make this five-point scale comparable to the expectation survey, scores were converted to a seven-point scale to match the expectation scale (maximum score of 5 on the evaluation survey converts to a score of 7, midpoint 3=4, and minimum 1=1, etc). This transformation of the data, a standard practice to enable the direct comparison of scales, was necessary to determine if there was a significant difference between expectations and the app experience using an independent *t* test [37-39].

To determine whether negative thinking had been reduced because of the app experience, days 1 and 7 app scores were compared with the exception of three participants who began using the app on day 2. A paired sample *t* test was then used to examine whether day 7 negative thinking scores as measured by the app survey were lower than day 1 scores. Pre and post MASC-2 total anxiety scores also were compared using a paired sample *t* test.

Qualitative data was analyzed using content analysis. Three black women between the ages of 21 and 25 years who were familiar with the culture and idiom of urban black girls transcribed the focus group audio digitals. A coding form was developed to assess each focus group question for each of the SUN sister circles. Coders read the transcripts, identified major themes, and included quotes that reflected the identified themes. In addition, coders determined if the themes identified related to the primary research questions of app feasibility and effectiveness. A consensus meeting was held with the data supervisor after coding was completed. Coders reviewed the themes and discussed any differences in their coding. Ultimately, the themes that emerged were the consensus of the three coders.

## Results

### Quantitative Evidence of Effectiveness

Descriptive statistics were calculated for relevant app expectation items that addressed the potential effectiveness of the app and are presented in Table 1. Scores ranged from 1 to 7 (7=strongly agree). The results for all expectation items were well above the median score (4) of the App Expectation Survey Likert scale. Also, the descriptive analysis indicated that the modes were all high (all scores of 7 or 6) and skewness was strongly negative for each item, indicating that girls’ expectations for the app were very high.

Descriptive statistics were calculated for each app expectation (mean and standard deviation) where scores could range from 1 to 7 (7=strongly agree): item 6, “It will be practical to pull out my phone and use the app when I feel stressed” (mean 6.2, SD 1.2); item 8, “I believe the app will be useful in calming my nerves for specific situations when I feel anxious” (mean 5.9, SD 1.4); item 9, “I expect that after using the app I will feel less anxious in general” (mean 5.9, SD 1.3), item 10, “I expect my theme song will make me feel powerful” (mean 6.1, SD 1.2)” and item 14, “I want to use the theme song app” (mean 5.7, SD 1.5). The results for all expectation items were well above the median score (4 of 7) of the Application Expectation Survey Likert scale. Also, the descriptive analysis indicated that the modes were all high (all scores of 7 or 6) and that skewness was strongly negative for each item further indicating that girls’ expectations for the app were very high.

The next question was to determine if these high expectations were maintained after experiencing the app. Independent *t* tests compared app expectation items 6 and 8 (these items were judged to be the best indicators of expectations of use and usefulness) with the session 7 evaluation question “I enjoyed recording my theme song” (mean 5.8, SD 1.5). The resulting *t* values were not significant. Thus, participants’ expectations of the app were high, and these expectations were maintained after actually experiencing the app.

**Table 1:** Descriptive statistics for selected App Expectation Survey items.

App Expectation Survey items	N	Participant responses (7=strongly agree)	
		Mean (SD)	Median
App will be practical to use	60	6.2 (1.2)	7.0
App will useful in calming nerves	59	5.9 (1.4)	6.0
Will feel less anxious using the app	59	5.9 (1.3)	6.0
The theme song will make me feel more powerful	60	6.1 (1.2)	6.0
Want to use the theme song app	59	5.7 (1.5)	6.0

A paired samples *t* test was used to determine whether negative thought was significantly lower at day 7 than day 1. Average negative thought scores on day 7 (mean 12.81, SD 4.22) were significantly lower than average negative thought scores on day 1 (mean 14.20, SD 4.10; *t*_31_=1.69, *P*=.05, Cohen *d*=0.30). On day 7, 42% of participants reported lower average negative thought scores versus day 1.

A paired sample *t* test compared girls’ pre-app to post-app total MASC anxiety T-scores to determine if a change in anxiety was evident. Results showed there was a significant decrease (*t*_38_=2.82, *P*=.004) in anxiety scores from preintervention (mean 56.28, SD 11.18) to postintervention (mean 53.21, SD 11.31) with a medium effect size (Cohen *d*=0.517).

### Qualitative Evidence of Effectiveness

During the guided discussions, four themes emerged related to the effectiveness of the BYOTS app: (1) differences in behavior and temperament, (2) promotes calmness, (3) helpfulness in stressful home situations, and (4) focused thinking via the SUN song

The theme “differences in behavior and temperament” relates to behavioral and attitude changes observed in participants by others as well as their own self-awareness of that change. These changes are illustrated in the following sample statements:

My mother has seen changes because I always use to catch an attitude with my sister and brother, but now I worries [sic] about myself and not everyone else.

I don’t cuss people out anymore.

A second theme identified reflects girls’ perceptions that the app “promoted calmness.” The following statement is an example of this theme:

When it tells you ‘you’re stressed’ at the end, it helps you think to yourself that you need to calm down.

The third theme that emerged was that girls found the app “helpful in home and familial situations.” The following statement exemplifies this theme:

I was arguing with my mom and sister. I ran up to my room, slammed the door. Then I thought, wait a minute, I can use my app. I listened to my song, went downstairs, and we worked it out.

The final theme that emerged centered on the ability of the app to focus one’s thinking. As noted earlier in this paper, when girls opened the app, it played the first seven bars of their theme song. The familiarity of the song allowed girls to focus on their thoughts. This theme is reflected in the statement:

When the S.U.N. song began to play, it calmed me down, and I could focus on my thinking.

## Discussion

### Principal Findings

Despite having higher levels of anxiety, and the consequences of that anxiety on adulthood, urban black girls are conspicuously absent from the mHealth app literature. To the best of our knowledge, this study is the first to develop an anxiety-related app for this population and evaluate its effectiveness. Our mixed methods results indicate that musical cognitive restructuring reduced their negative thinking. Indeed, negative thoughts decreased from day 1 to day 7. Changes in thinking and behavior were noted by the girls themselves, their friends and family, and by the data collected within the app. Anxiety was also reduced pre-post intervention.

Our findings suggest that an mHealth app can be an important tool in anxiety intervention with this population. It should be noted that BYOTS is not a stand-alone app but is used in conjunction with SUN culturally relevant intervention. Therefore, although we are able to say that the app played a role in the reduction of anxiety, we cannot say that it was solely responsible for the reduction in anxiety. We have designed a study in which we compare three groups (app only, intervention only, app-augmented intervention); this will allow us to further understand the app’s effectiveness in reducing anxiety.

Not only the girls themselves, but their families and friends, noted the change in thinking and attributed that change to using the app. This attribution suggests that we take a closer look at the mechanisms underlying musical cognitive restructuring to determine what components are facilitating the change. Is it the lyrics, the rhythm, the tempo, the fact that it is contained within an app, the self-monitoring aspect, or some combination of these factors? A closer look at key elements would allow us to gain further understanding as to why BYOTS works. To this end, we recently completed a study examining the lyric, rhythm, and tempo component of participants’ theme songs. Findings should be available soon.

As a result of the observed changes, family members—particularly mothers—expressed interest in using the app. Although BYOTS is designed for adolescent females, the possibility exists that the app may also be an effective tool for adult black women.

The emergent theme “focused thinking via the song” was an unexpected finding. The SUN intervention has a theme song that is used to close each session. When the BYOTS app is opened, the first seven bars of the song are heard. It appears that for some participants, hearing the intervention theme song was a cue to self-monitor thoughts [40], the first step in cognitive restructuring. In subsequent planned studies, we will further explore this finding.

Girls’ expectations of the app matched their experience with the app. Comparing expectations and experience is a standard procedure in intervention research; however, this result holds added meaning for our sample. As part of the focus groups, girls shared their skepticism as to whether the app would meet their expectations. Participants indicated that they had worked with other researchers where what was expected had not matched the actual experience. They expressed great delight “it really worked” regarding their app experiences.

A frequent question that arises in our work is “what about adolescent black boys?” Clearly, a need exists to develop an anxiety mHealth app for this group. Given the robust finding that boys respond to the tempo and rhythm to elevate mood [26], rather than the lyrics of a song, in its current iteration BYOTS may not be a good fit for black males. We plan to undertake a series of focus group studies to determine if and how the app should be modified for this population.

### Limitations

This study represents an open trial and a first step in empirically evaluating the BYOTS app. The study was also limited to an urban, low-income population. However, black girls are heterogenous and encompass all socioeconomic statuses and reside in various types of locales. Subsequent studies would extend the work to black girls of various socioeconomic statuses residing in urban, rural, and suburban locales. Already, we are conducting an open trial with girls residing in a small town, and developing and laying the groundwork for a multisite randomized controlled trial.

For research purposes, use of the app was limited to a 1-week period. In subsequent studies, we plan to extend app use to 1 month. This will allow us to gather further data, including frequency of unprompted use, time of day most used, and so on.

### Conclusions

The BYOTS app appears to be a useful tool in reducing negative thinking among urban, low-income, middle-school black girls. Our findings add to the knowledge base as to how mHealth can be used with underserved and underresearched adolescents. As elucidated in our Discussion, these results provide direction for subsequent research with this mHealth app.

## Abbreviations

BYOTS: Build Your Own Theme Song
MASC-2: Multidimensional Anxiety Scale-2
SUN: Sisters United Now
